# An AI-Driven Dual-Spectral Vision–Language Sensing Framework for Intelligent Agricultural Phenotyping

**DOI:** 10.3390/s26072045

**Published:** 2026-03-25

**Authors:** Lei Shi, Zhiyuan Chen, Chengze Li, Yang Hu, Xintong Wang, Haibo Wang, Yihong Song

**Affiliations:** 1 China Agricultural University, Beijing 100083, China; 2 National School of Development, Peking University, Beijing 100871, China

**Keywords:** artificial intelligence-driven sensing, multimodal sensor fusion, spectral–vision perception, intelligent sensor systems, high-throughput intelligent inspection

## Abstract

Seed varietal purity and physiological viability are critical determinants of crop yield and quality. However, non-destructive assessment faces significant challenges in fine-grained variety discrimination and the perception of internal defects. This study proposes S3-Net, an AI-driven multimodal sensing framework that integrates vision–language alignment with dual-spectral sensor fusion for autonomous seed quality evaluation. We introduce a Knowledge–Vision Alignment (KVA) module that incorporates encyclopedic morphological descriptions to guide feature learning, significantly enhancing few-shot generalization. Complementarily, a Dual-Spectral Fusion (DSF) module combines high-resolution RGB textures with penetrative Short-Wave Infrared (SWIR) sensing to jointly characterize external and internal traits. Experimental results on a custom multimodal dataset of 6000 samples across 12 crop categories demonstrate that S3-Net achieves 96.9% accuracy for species identification and 95.8% for viability detection. Notably, S3-Net outperforms ResNet-50 by 40.3% in extreme 1-shot scenarios. With a stable inference throughput of 95 fps, the system meets the high-throughput demands of industrial-scale applications, providing a robust and efficient solution for intelligent agricultural phenotyping.

## 1. Introduction

Crop seeds serve as the fundamental carriers of agricultural production, with their varietal purity and physiological activity acting as determinant factors for crop yield and quality. With the rapid advancement of precision agriculture and automated breeding technologies, the establishment of efficient, non-destructive, and high-throughput systems for seed phenotyping and quality assessment is of significant importance for ensuring germplasm resource security and enhancing agricultural production efficiency. In traditional agricultural production and research, seed screening has predominantly relied on manual visual inspection; this approach is not only inefficient but also susceptible to subjective factors, leading to inconsistent discrimination standards. As early as 2000, computer vision technology was introduced by Majumdar et al. for grain seed classification, validating the feasibility of automated image analysis in agricultural detection [1]. Nevertheless, under non-destructive conditions, simultaneous achievement of precise identification of fine morphological differences among closely related varieties and effective assessment of internal physiological states remains a significant challenge within the fields of agricultural engineering and machine vision.

Vision-based seed identification methods have primarily evolved through two stages: manual feature engineering and deep representation learning. Early research focused on the extraction of explicit physical features, such as morphological parameters, color histograms, and texture co-occurrence matrices, combined with Support Vector Machines (SVMs) or Artificial Neural Networks (ANNs) to construct classification models [2,3,4]. Although these methods performed well on specific datasets, handcrafted features often fail to meet practical application requirements due to insufficient discriminative power when addressing fine-grained classification tasks with high inter-class similarity [5]. In recent years, Convolutional Neural Networks (CNNs), relying on powerful end-to-end feature extraction capabilities, have achieved performance significantly superior to traditional methods in seed image classification tasks [6]. However, the performance of supervised deep learning models is highly dependent on large-scale and high-quality labeled data. In the agricultural domain, acquiring expert-level labeled data containing rich varieties and diverse physiological states is costly, and sample distributions often exhibit long-tail characteristics; this limits the generalization ability of models regarding new varieties or rare disease categories lacking samples [7]. To address the issues of sample scarcity and annotation difficulty, self-supervised learning and vision–language pre-training models (such as CLIP) have provided new solution paradigms [8,9]. These methods demonstrate superior feature transfer capabilities by mining the intrinsic structural features of data or utilizing semantic supervision information from natural language. For instance, self-supervised clustering methods were utilized by Güldenring et al. to pre-train on unlabeled agricultural images, effectively improving classification accuracy under low-sample conditions [10]; meanwhile, Zhou et al. achieved efficient zero-shot identification of crop diseases by introducing text descriptions generated by Large Language Models (LLMs) and utilizing CLIP [11,12]. Despite these advancements, problems regarding domain feature distribution mismatch and semantic adaptability remain to be resolved when transferring general-domain pre-trained models to agricultural microscopic vision tasks [13,14].

Beyond the aforementioned data-level challenges, single visible light imaging modalities possess physical limitations in detecting the internal physiological characteristics of seeds. Seed germination rates and vigor are often affected by factors such as internal mildew, insect-induced cavities, or endosperm necrosis; these internal defects are typically obscured by intact seed coats and cannot be directly observed via RGB images [15]. Consequently, the introduction of penetrating infrared imaging or hyperspectral technology has become an inevitable choice for non-destructive testing [16]. Research by Liu et al. confirmed that near-infrared spectroscopy can effectively capture spectral response differences caused by changes in internal chemical components of seeds, thereby achieving precise grading of internal mildew in in-shell sunflower seeds [17]. To balance the high-resolution texture features of visible light with the internal perspective advantages of infrared light, multi-modal image fusion technology has gradually become a research hotspot [18]. Existing deep fusion algorithms attempt to construct joint feature representations via CNN or Transformer architectures to enhance detection comprehensiveness [19,20,21]. However, in seed microscopic detection scenarios, spatial resolution differences between heterogeneous sensors lead to registration difficulties [22,23]. Furthermore, existing fusion mechanisms struggle to effectively maintain the detailed texture structure of visible light images while preserving infrared thermal anomaly features, constituting a technical barrier to high-precision joint discrimination.

Despite the progress in seed phenotyping, existing methods still face two primary bottlenecks. First, traditional RGB-based deep learning models are limited to surface-level morphological features and cannot perceive internal physiological status (e.g., vigor or latent lesions). Second, while Hyperspectral Imaging (HSI) provides rich spectral information for internal sensing, its high data dimensionality often leads to prohibitive computational costs and low throughput (typically <10 fps), making it unsuitable for real-time industrial sorting lines that require processing hundreds of seeds per second. Furthermore, most current multimodal frameworks are purely data-driven and “knowledge-blind”; they neglect the rich botanical encyclopedic priors used by human experts, resulting in poor generalization in few-shot scenarios and limited discriminative power for visually near-identical varieties.

To bridge these gaps, this paper proposes S3-Net, a unified identification framework that achieves a paradigm shift from dual-modal spectral fusion to “Vision-Language-Spectral” ternary synergy. Unlike existing multimodal architectures that merely concatenate heterogeneous sensors, S3-Net introduces three core innovations: (1) Semantic-Guided Discrimination: By incorporating botanical encyclopedic knowledge through a Knowledge–Vision Alignment (KVA) module, the framework utilizes high-level morphological descriptors as “semantic anchors” to resolve fine-grained ambiguities that baffle purely visual or spectral models. (2) High-Throughput Internal-External Sensing: We employ an optimized Dual-Spectral Fusion (DSF) strategy that pairs high-resolution RGB textures with penetrative SWIR signals, achieving a balance between deep physiological perception and industrial-scale throughput (95 fps). (3) Knowledge-Driven Robustness: By leveraging vision–language pre-training, S3-Net demonstrates superior generalization in data-scarce (few-shot) scenarios, effectively digitizing expert botanical expertise into the automated inspection workflow. Finally, by integrating this framework with a continuous scanning device, we demonstrate a practical, full-coverage inspection system capable of high-throughput recognition in real-world agricultural production. The main contributions of this paper are summarized as follows:

1. An AI-driven multimodal intelligent sensing system for high-throughput seed inspection is developed. A conveyor-based dual-camera (RGB–SWIR) acquisition device is seamlessly integrated with the proposed semantic–spectral perception network, forming a real-time sensing–analysis pipeline. The system enables synchronized multi-spectral data acquisition, autonomous decision-making, and industrial-scale deployment, establishing a practical intelligent sensor framework for agricultural production.
2. A knowledge–vision alignment (KVA) module is proposed to enhance semantic-aware sensing capability. By constructing a cross-modal mapping mechanism between visual sensor features and encyclopedic textual semantics, domain knowledge is injected into the perception space through attention-based alignment. This design reduces dependence on large-scale labeled data and significantly improves robustness under few-shot and zero-shot conditions.
3. A dual-spectral fusion (DSF) module is designed to achieve comprehensive physical–semantic perception. Through attention-guided infrared refinement and multi-scale visible feature aggregation, the penetrative sensing capacity of SWIR imaging is synergistically combined with high-resolution RGB texture representation. This unified framework enables simultaneous fine-grained variety discrimination and non-destructive viability detection within a single AI-driven sensing architecture.
4. From both technical and agricultural–economic perspectives, the proposed framework improves seed screening efficiency, reduces misclassification-induced yield losses, and enhances germplasm resource management. By enabling accurate, high-throughput, and data-efficient quality assessment, the system supports precision agriculture practices and contributes to yield stability and input–output optimization across the agricultural supply chain.

## 2. Materials and Methods

### 2.1. Research Overview

**Figure 1:**
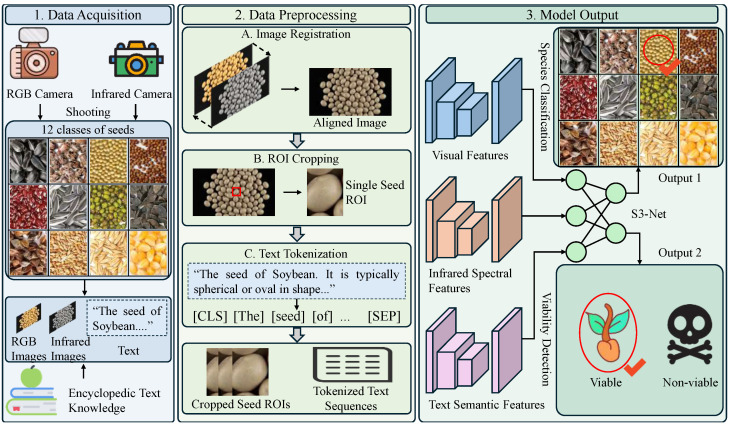
Schematic overview of the proposed experimental framework. The workflow is organized into three logical stages: (1) Data Acquisition: Utilizing a dual-camera system (RGB and Infrared) alongside an agricultural knowledge base to construct a high-quality multi-modal dataset covering 12 crop categories. (2) Data Preprocessing: Implementing high-precision image registration, single-seed ROI cropping, and text tokenization to standardize heterogeneous inputs. (3) Model Output: The S3-Net architecture hierarchically fuses visual features, infrared spectral features, and text semantic features to simultaneously achieve fine-grained species classification (Output 1) and seed viability detection (Output 2).

To achieve the non-destructive and precise assessment of crop seeds, we constructed a systematic research framework integrating multi-modal hardware acquisition, standardized data processing, and intelligent modeling, as visually illustrated in Figure 1. The workflow follows a rigorous three-stage pipeline. In the data acquisition phase, a custom-built dual-sensor system captures synchronized RGB and Short-Wave Infrared (SWIR) images, while simultaneously curating a corpus of encyclopedic text descriptions for 12 distinct seed categories. Subsequently, the data preprocessing stage addresses signal heterogeneity through pixel-level image registration, adaptive ROI cropping, and Transformer-based text tokenization (embedding [CLS] and [SEP] tokens), ensuring strict spatial and semantic alignment across modalities. Finally, these standardized data triplets are fed into the S3-Net Model, which extracts and dynamically fuses visual textures, infrared spectral signatures, and semantic representations. This unified architecture ultimately outputs dual predictions: fine-grained species classification and non-invasive viability detection (Viable vs. Non-viable), realizing a comprehensive evaluation of seed quality.

### 2.2. Data Acquisition

To construct a seed dataset possessing high regional representativeness and multimodal characteristics, data acquisition for this experiment was centrally conducted during the harvest season from September to October 2023 at the experimental fields of the Hetao Irrigation District Academy of Agricultural Sciences in Bayannur, Inner Mongolia, China (40°45′ N, 107°24′ E). As a renowned commercial grain and sunflower production base in China, Bayannur possesses unique advantages in germplasm resources. Twelve types of typical crop seeds widely cultivated in this region were selected as research objects, covering oil crops, grain crops, and legumes. Specifically, the species and their respective cultivars included: edible sunflower (*Helianthus annuus* L., cv. SH363), oil sunflower (*Helianthus annuus* L., cv. G101), maize (*Zea mays* L., cv. Jinshan 27), wheat (*Triticum aestivum* L., cv. Yongliang 4), soybean (*Glycine max* (L.) Merr., cv. Zhonghuang 13), red kidney bean (*Phaseolus vulgaris* L., cv. British Red), mung bean (*Vigna radiata* (L.) R. Wilczek, cv. Weilv 4), sorghum (*Sorghum bicolor* (L.) Moench, cv. Jiazhou 5), buckwheat (*Fagopyrum esculentum* Moench, cv. Neiqiao 1), oat (*Avena sativa* L., cv. Mengyan 1), castor bean (*Ricinus communis* L., cv. Zibo 5), and pumpkin seed (*Cucurbita moschata* Duchesne, cv. Jinguayuan 1). To simulate complex physiological states of seeds in authentic agricultural scenarios, sample collection encompassed not only healthy seeds but also a substantial number of low-vigor samples obtained through natural and artificial aging. The natural aging (NA) process involved storing seeds in a conventional ventilated warehouse for 12 months under ambient conditions (average temperature 18±5 °C, relative humidity 55±10%), allowing for the gradual decline of vigor through spontaneous lipid oxidation and metabolic exhaustion.

Artificial accelerated aging (AA) was conducted following the standard protocol of the International Seed Testing Association (ISTA) [24]. Specifically, seeds were placed in a single layer on a stainless steel mesh tray suspended over 50 mL of distilled water inside a sealed aging box (12×12×5 cm). These boxes were then maintained in an intelligent artificial aging chamber at 45 °C and 95% relative humidity for 72 h. This methodology for inducing vigor deterioration is widely recognized and adopted in seed science for simulating long-term storage effects in a compressed timeframe.

All collected raw samples underwent a standardized cleaning procedure prior to imaging. This process involved the removal of physical impurities (such as glumes, stems, and inert matter) and broken seeds using a laboratory air-suction cleaner. Subsequently, the seed surfaces were gently wiped with a fine-bristle brush to remove adhering dust and soil, ensuring that the acquired RGB and SWIR spectral signatures strictly represented the seed’s own morphological and physiological traits. These steps resulted in the final selection of 6005 representative single-seed samples.

Image data acquisition was completed within a specially constructed darkroom to eliminate interference from ambient stray light. Seeds are conveyed in a single-layer flow channel at a rate of 500 seeds per second. We implemented a two-stage protocol to separate the dataset construction phase from the system evaluation phase. To obtain ground truth values, seeds were arranged on local grid segments rather than randomly distributed. Each seed was assigned a unique spatial coordinate, synchronized between the imaging system and the germination tray, achieving a precise one-to-one correspondence between multimodal images and subsequent 14-day germination results. The reported throughput refers to the system’s sustained operational capability during the inference and industrial deployment phases, validated using pre-sorted samples after model training. The imaging system consisted of visible light and short-wave infrared (SWIR) imaging units, fixed vertically 20 cm above the stage. Specifically, the visible light imaging unit employed a Hikvision MV-CA050-20GC industrial camera (Hangzhou Hikrobot Co., Ltd.; Hangzhou, China) equipped with a 16 mm fixed-focus lens, offering a resolution of 2448×2048, to capture high-definition apparent texture and color information of the seeds. The infrared imaging unit utilized a Xenics Bobcat-320 Short-Wave Infrared (SWIR) camera (Xenics; Leuven, Belgium), with a detection band covering 900 nm to 1700 nm and a resolution of 320×256. The 900–1700 nm SWIR band was strategically selected as it encompasses the “biological optical window,” where the scattering coefficient of woody seed coats (composed primarily of lignin and cellulose) is significantly lower than in the visible spectrum. This reduction in Mie scattering allows IR photons to penetrate deeper into the endosperm and embryo regions. Specifically, the system targets the first and second overtones of O-H and C-H stretching vibrations, which are sensitive to internal moisture distribution and lipid degradation. To ensure consistency in dual-band imaging, the system adopted a set of halogen tungsten lamps covering the full visible to near-infrared spectrum as the light source, used in conjunction with a soft light cover to form uniform diffuse illumination, thereby effectively avoiding specular reflection highlights caused by seed surface curvature. A black light-absorbing flannel with a reflectivity of less than 2% was selected as the background board to simplify subsequent background segmentation. During the acquisition process, microsecond-level synchronous exposure was achieved between the two cameras via hardware trigger signals, ensuring strict temporal alignment of the RGB and infrared images for the same seed. To quantitatively characterize the data composition of the multimodal sensing framework, the sources and scales of data acquired from each sensor were systematically summarized, as presented in Table 1. The RGB vision sensor and the short-wave infrared sensor respectively captured modality-specific image data, while auxiliary sensing units synchronously recorded imaging environment parameters and seed conveyance states. Together, these heterogeneous sensing data streams constitute a comprehensive intelligent perception data foundation for subsequent model training and evaluation.

Data labeling strictly adhered to agronomic standards, encompassing two dimensions: variety and viability. Variety labels were manually identified and confirmed by agricultural experts with over ten years of experience. Viability labels were obtained through destructive verification in accordance with the International Rules for Seed Testing (ISTA Rules). Following image acquisition, all samples were numbered and placed in a constant temperature and light incubator for standard germination testing (Paper Bed Method). After a cultivation period of 7 to 14 days (depending on the crop species), seed vigor was determined based on seedling growth status (e.g., radicle length, cotyledon morphology). If a seed failed to germinate or produced a malformed seedling, it was labeled as Non-viable; otherwise, it was labeled as Viable. Concurrently, to support the construction of the Knowledge–Vision Alignment (KVA) module, a complementary seed encyclopedia text corpus was established. Textual data were crawled from the Chinese Crop Germplasm Resources Information Network and the agricultural section of Wikipedia, and were manually revised with reference to local agricultural technology extension manuals in Bayannur. For each seed category, natural language descriptions covering morphological characteristics (e.g., oval shape, yellow seed coat), taxonomic information, and growth habits were compiled, generating approximately 5 to 10 high-quality image-text pairs per category. Specific statistical information regarding the dataset is presented in Table 2.

### 2.3. Data Preprocessing

Raw multimodal data acquired from sensors exhibits issues such as mismatched resolutions, background redundancy, and unstructured text, rendering it unsuitable for direct input into neural networks for training. Consequently, a standardized preprocessing pipeline was designed, primarily comprising four stages: cross-modal spatial registration, region of interest (ROI) extraction, synchronized data augmentation, and text serialization.

First, to address the field-of-view deviation and resolution discrepancy between the visible light camera (2448×2048) and the infrared camera (320×256), high-precision geometric correction is essential to achieve pixel-level alignment. Using images of a 10×7 checkerboard calibration pattern captured during the acquisition phase, the homography matrix between the two sensors was calculated by detecting corner coordinates. Utilizing this matrix, bilinear interpolation was employed to map the infrared image into the coordinate system of the visible light image. This process eliminates parallax resulting from differing sensor mounting positions, ensuring strict spatial coincidence of seed edges and textures. The corrected infrared images were upsampled to match the resolution of the visible light images, thereby constructing four-channel RGB-IR aligned data.

Second, to eliminate interference from the black background during feature extraction and reduce computational redundancy, threshold-based Region of Interest (ROI) extraction was implemented. High-resolution RGB images were converted to grayscale, and Otsu’s binarization was applied to adaptively calculate thresholds and generate binary masks. Morphological closing operations were subsequently performed to fill potential holes within the seeds. Based on these masks, the minimum bounding box for each seed was calculated and expanded by 10 pixels to preserve complete edge information. Using these coordinates, both the RGB images and the registered infrared images were simultaneously cropped. The resulting single-seed images were uniformly resized to 224×224 pixels. During this process, all pixel values were normalized to the [0,1] interval and underwent Z-score standardization based on the mean and standard deviation of the ImageNet dataset to accelerate model convergence.

To prevent model overfitting on limited samples and enhance robustness to variations in imaging environments, an online data augmentation strategy was introduced during the training phase. Considering the consistency constraints of dual-modal data, all geometric transformations were applied synchronously to both RGB and infrared images. Specific augmentation operations included random horizontal and vertical flipping (probability p=0.5), random rotation (angle range ±15°), and random scaling (scale factor 0.8∼1.2). Furthermore, modality-specific augmentations were introduced to address distinct physical characteristics: slight color jittering (brightness and contrast variation range ±10%) was applied to RGB images to simulate illumination fluctuations, while zero-mean Gaussian noise (σ=0.01) was injected into infrared images to simulate the inherent electronic noise of thermal imaging sensors, thereby improving the model’s adaptability to real-world agricultural scenarios.

Finally, regarding the unstructured encyclopedic text descriptions of seeds, a Transformer-based preprocessing scheme was adopted. Text was first processed through case conversion and regularization to remove special symbols and excess spaces. Subsequently, a pre-trained tokenizer was utilized to segment natural language sentences into sub-word sequences. To accommodate the input requirements of the Transformer encoders within the KVA module, special tokens ‘[CLS]’ and ‘[SEP]’ were added to the beginning and end of the sequences, respectively. The maximum sequence length was uniformly truncated or padded to 77 tokens. Through these procedures, the text data were converted into standard Token ID matrices and Attention Mask matrices, which, together with the image data, constitute the input triplets for the model.

### 2.4. Proposed Method

#### 2.4.1. S3-Net Overview

**Figure 2:**
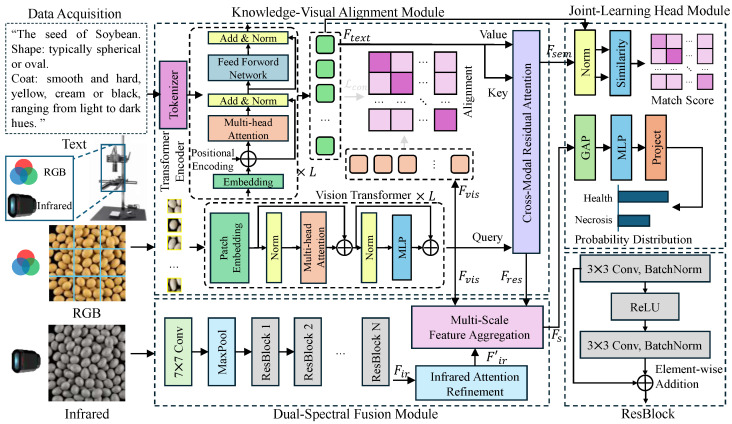
Overview of the overall architecture of the proposed Semantic–Spectral Seed Network (S3-Net). The framework primarily comprises three components: the knowledge–vision alignment (KVA) module, the dual-spectral fusion (DSF) module, and the joint learning prediction head (JLH). By jointly optimizing the image-text semantic matching task and the multi-spectral viability detection task, the model achieves simultaneous and precise discrimination of seed varieties and internal viability.

To overcome the limitations of single-modal sensing and heavy annotation reliance, we propose the Semantic–Spectral Seed Network (S3-Net). This framework mimics human expert cognition by integrating botanical encyclopedic knowledge as semantic priors with multi-spectral physical perception. As illustrated in Figure 2, S3-Net is an end-to-end multimodal architecture comprising three core modules: Knowledge–Vision Alignment (KVA), Dual-Spectral Fusion (DSF), and Joint Learning Prediction Head (JLH). The KVA module aligns visual features with text embeddings via cross-modal attention, injecting high-level morphological concepts to enhance few-shot generalization. Building on this, the DSF module employs a ternary fusion strategy to integrate these semantically-enhanced features with high-resolution RGB textures and penetrative SWIR signals. This synergy captures both internal physiological states and external morphological traits. Finally, the JLH performs joint optimization, outputting variety matching scores based on cosine similarity and viability probabilities through deep feature fusion. This unified pipeline enables precise, simultaneous assessment of seed variety and internal quality.

#### 2.4.2. Scanning Imaging Hardware Device

**Figure 3:**
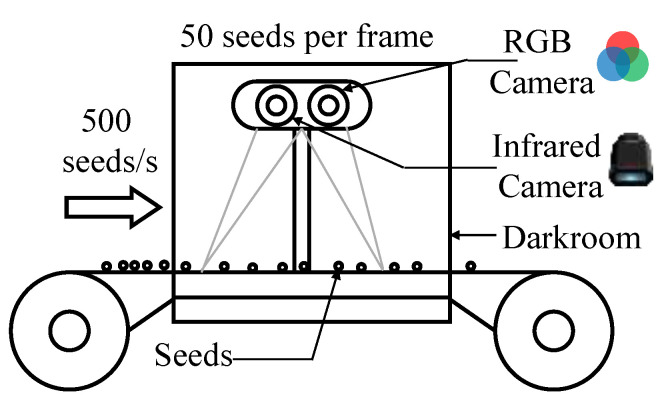
Schematic diagram of the scanning imaging hardware device. The diagram illustrates the main structural components and their spatial arrangement, as well as the key parameter settings during system operation.

To facilitate practical deployment of the proposed method in industrial scenarios, we modeled a complete system for real-time seed discrimination and identification based on the continuous seed flow paradigm of conveyor-based pipelines, as illustrated in Figure 3. The overall setup consists of a conveyor belt and an enclosed darkroom, within which a camera mounting structure is installed to accommodate two parallelly aligned imaging units, namely a visible-light camera and an infrared camera. Seeds are distributed on the conveyor belt at a controlled density to ensure that each field of view contains approximately 50 seeds per frame. The conveyor belt operates at a throughput of 500 seeds per second, while the two cameras synchronously perform continuous image acquisition within the darkroom to guarantee full coverage of all seeds passing through the scanning region. The fields of view of the two cameras are consistently aligned to enable precise multimodal correspondence, and the captured visible and infrared image streams are transmitted to the processing unit via high-speed interfaces for real-time semantic–spectral analysis by the proposed S3-Net.

Under industrial operating conditions, it is necessary to quantitatively derive the minimum camera frame rate and model inference throughput required to guarantee continuous and lossless processing of the seed stream. Let $R_{s}$ denote the seed flow rate (seeds/s), $N_{f}$ the average number of seeds captured per frame, and $F_{c}$ the required camera frame rate (frames/s). To ensure full coverage of all seeds passing through the imaging region, the minimum camera frame rate is determined as

(1) $$F_{c}=\frac{R_{s}}{N_{f}}$$

Furthermore, to enable real-time processing without buffering or delay, the minimum model inference throughput $T_{m}$ (fps) must match the camera acquisition rate, yielding

(2) $$T_{m}=F_{c}$$

These expressions define the lower-bound operational requirements of the system. In the experimental configuration, where approximately 50 seeds are captured per frame and the seed flow rate is set to 500 seeds/s, the minimum required frame rate and inference throughput are both 10 fps. Experimental results demonstrate that the proposed S3-Net achieves a stable throughput of up to 95 fps even under peak-load conditions, providing a substantial performance margin over the minimum requirement and confirming its suitability for industrial-scale deployment.

#### 2.4.3. Knowledge–Vision Alignment Module

**Figure 4:**
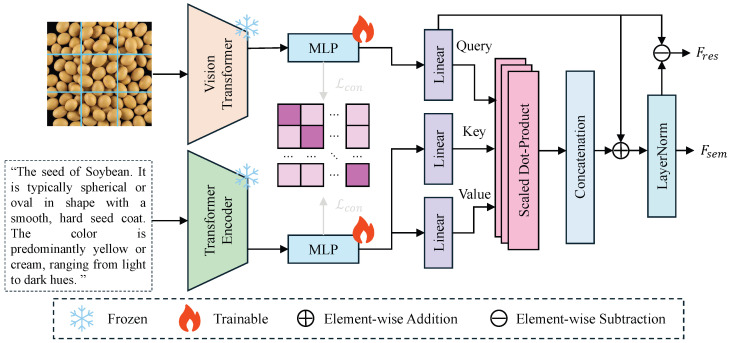
Detailed architecture of the knowledge–vision alignment module. The illustration demonstrates how the module utilizes parameter-frozen pre-trained encoders to extract visual and textual features, respectively, and maps heterogeneous modalities into a unified feature space via the contrastive loss $L_{con}$. The details on the right display the process by which the Cross-Modal Residual Attention mechanism injects textual semantics into visual features to generate the enhanced feature $F_{sem}$.

To address the limitations of traditional seed recognition methods, which rely solely on visual appearance features and struggle with high inter-class similarity and scarce training samples, the knowledge–vision alignment (KVA) module is designed, as illustrated in Figure 4. By introducing semantic information from seed encyclopedic knowledge into the visual representation space, this module utilizes rich high-level semantic concepts contained in natural language descriptions (e.g., spherical, yellow seed coat) to assist the model in interpreting image content. The KVA module adopts a dual-stream architecture to independently extract visual features from images and semantic features from texts. Subsequently, Multi-Layer Perceptrons (MLPs) are employed to map both feature sets into a unified feature space for alignment. On this basis, a cross-attention mechanism is utilized to inject textual semantics as prior knowledge into visual features. This process generates a semantically enhanced seed feature representation, denoted as $F_{sem}$, which significantly improves the generalization capability of the model in few-shot or zero-shot scenarios while preserving visual perception abilities.

Specifically, given an input seed image $I\in R^{H\times W\times 3}$ and its corresponding encyclopedic text description *T*, the computational process is divided into three stages: feature extraction and projection, cross-modal alignment loss calculation, and semantic injection. First, to leverage the general knowledge of large-scale pre-trained models and reduce computational overhead, a parameter-frozen Vision Transformer ($E_{v}$) and a Transformer Encoder ($E_{t}$) are adopted as the backbone networks. Following encoding, the image and text yield initial feature representations $f_{v}\in R^{D_{v}}$ and $f_{t}\in R^{D_{t}}$, respectively. Due to the inconsistency in dimensions between visual and textual features, two trainable Multi-Layer Perceptrons, ${\mathrm{MLP}}_{v}$ and ${\mathrm{MLP}}_{t}$, are introduced as projection heads to map both into a common embedding space of dimension *d*:

(3) $$z_{v}={\mathrm{MLP}}_{v}\left(f_{v}\right),\;z_{t}={\mathrm{MLP}}_{t}\left(f_{t}\right)$$

where $z_{v},z_{t}\in R^{d}$ represent the aligned feature vectors.

To constrain the consistency between visual and semantic features and ensure that semantically related image-text pairs are as close as possible in the feature space, Symmetric Contrastive Loss is introduced. Assuming a batch contains *N* image-text pairs, for the *i*-th sample, the normalized similarity between its image feature $z_{v,i}$ and text feature $z_{t,i}$ is calculated as follows:

(4) $$\begin{array}{cc} L_{\mathrm{con}}=-\frac{1}{2N}\sum_{i=1}^{N} & (\log\frac{\exp(z_{v,i}\cdot z_{t,i}^{⊤}/\tau )}{\sum_{j=1}^{N}\exp\left(z_{v,i}\cdot z_{t,j}^{⊤}/\tau \right)}\\ & \;+\log\frac{\exp(z_{t,i}\cdot z_{v,i}^{⊤}/\tau )}{\sum_{j=1}^{N}\exp\left(z_{t,i}\cdot z_{v,j}^{⊤}/\tau \right)}) \end{array}$$

where · denotes the dot product operation, and τ is a learnable temperature coefficient. This loss function explicitly pulls positive pairs closer while pushing negative pairs apart.

Finally, semantic injection is realized through a cross-attention mechanism. The visual feature $z_{v}$ serves as the Query, while the text feature $z_{t}$ serves as the Key and Value. Corresponding projection vectors are generated through linear transformation matrices $W_{Q},W_{K},W_{V}\in R^{d\times d_{k}}$. To integrate fine-grained semantic information, Scaled Dot-Product Attention is utilized to calculate the image-text affinity matrix, which is then used for a weighted summation with *V*. Subsequently, residual connections and Layer Normalization (LayerNorm) are introduced to prevent gradient vanishing and accelerate convergence, ultimately outputting the semantically enhanced feature $F_{sem}$:

(5) $$\begin{array}{cc} Q=z_{v}W_{Q},\;K & =z_{t}W_{K},\;V=z_{t}W_{V}\\ \mathrm{Attention}(Q,K,V) & =\;\mathrm{softmax}\left(\frac{QK^{⊤}}{\sqrt{d_{k}}}\right)V\\ F_{sem}=\mathrm{LayerNorm} & \;(z_{v}+\mathrm{Attention}\left(Q,K,V\right)) \end{array}$$

Furthermore, to preserve the differential information between the original visual features and the enhanced features, the module additionally outputs a residual feature $F_{res}=F_{sem}-z_{v}$ to assist in subsequent discrimination tasks.

By freezing the weights of the pre-trained backbone networks, this module effectively preserves the general feature extraction capabilities learned from massive datasets, thereby avoiding the risk of overfitting potentially caused by fine-tuning on small-sample agricultural data. To further prevent the model from degenerating into rote memorization of text tokens as simple category labels—a risk potentially elevated by the small-scale text dataset—the KVA module leverages the descriptive nature of the input. Unlike simple integer labels, the 5–10 encyclopedic descriptions per category contain explicit morphological and taxonomic logic (e.g., “conical shape,” “striated pattern”). These descriptions act as semantic anchors that activate universal physical concepts already embedded within the high-dimensional manifold of the frozen pre-trained encoders. The cross-attention mechanism facilitates fine-grained attribute-level grounding, where visual feature patches are aligned with specific descriptive tokens rather than holistic class IDs. This ensures that the model learns the underlying visual-physical logic of seed traits, which is inherently generalizable across species, rather than performing trivial one-to-one mapping. Furthermore, the symmetric contrastive loss $L_{con}$ serves as a cross-modal regularizer, forcing the projected features to converge on a shared semantic space that represents biological attributes, effectively preventing the collapse into simple label-token associations. At the feature interaction level, the introduction of the contrastive loss $L_{con}$ plays a crucial regularization role; it explicitly reduces the distributional distance between heterogeneous modalities, ensuring high consistency between visual features and text descriptions within the semantic space. Furthermore, the cross-attention mechanism achieves fine-grained feature fusion by calculating the image-text affinity matrix. This ensures that the final feature representation $F_{sem}$ not only retains the intuitive geometric structure of the seeds but also successfully incorporates highly discriminative encyclopedic semantic information. This integration of form and meaning lays a solid foundation for subsequent high-precision classification.

#### 2.4.4. Dual-Spectral Fusion Module

**Figure 5:**
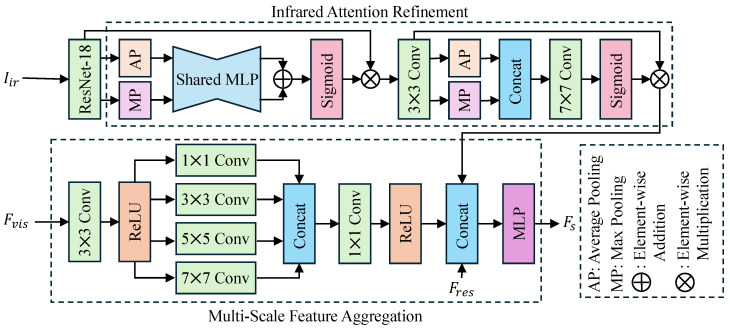
Diagram of the multi-level feature processing mechanism within the dual-spectral fusion module. The upper branch represents the Infrared Attention Refinement unit, which utilizes channel–spatial attention mechanisms to suppress thermal imaging noise. The lower branch represents the Multi-Scale Feature Aggregation unit, which employs parallel convolutions to capture visible light textures of varying granularities. Finally, the module adopts a ternary fusion strategy, combining infrared features, visible light features, and semantic residual features ($F_{res}$) to output the viability discrimination feature.

To overcome the limitations of single visible light modalities in detecting latent internal characteristics of seeds (such as mildew and hollow shells) and to fully leverage the complementary advantages of multi-source information, the dual-spectral fusion module (DSF) is proposed. As illustrated in Figure 5, the design intent of this module is to construct a multi-dimensional perception space that can precisely localize internal thermal discrepancies via infrared spectroscopy while simultaneously capturing rich multi-scale textures using visible light. Furthermore, residual semantic information from the KVA module is fused to enhance discriminative robustness. Macroscopically, the DSF module is constituted by two parallel branches: the Infrared Attention Refinement branch and the Multi-Scale Feature Aggregation branch. The former utilizes a hybrid attention mechanism to suppress infrared background noise, whereas the latter extracts apparent details through multi-receptive field convolutions. Ultimately, the module adopts a ternary feature fusion strategy, jointly modeling infrared features, visible light features, and semantic residual features ($F_{res}$) to output the high-precision viability discrimination feature $F_{s}$.

Specifically, the computational process comprises three steps: attention enhancement, multi-scale extraction, and ternary fusion. First, in the infrared branch, based on the feature $F_{ir}$ extracted from the input image $I_{ir}$ via ResNet-18, the Channel and Spatial Attention Mechanism (CBAM) is introduced to reinforce the perception of defective regions. Channel attention aggregates spatial information through parallel Max Pooling (MaxPool) and Average Pooling (AvgPool), generating channel weights $M_{c}$ via a shared MLP and Sigmoid activation. Spatial attention generates spatial weights $M_{s}$ using a 7×7 convolution after pooling in the channel dimension. The refined infrared feature $F_{ir}^{att}$ is calculated as follows:

(6) $$\begin{array}{cc} M_{c}\left(F_{ir}\right) & =\sigma (\mathrm{MLP}\left(\mathrm{AvgPool}\left(F_{ir}\right)\right)+\mathrm{MLP}\left(\mathrm{MaxPool}\left(F_{ir}\right)\right))\\ F_{ir}^{\prime } & =M_{c}\left(F_{ir}\right)⊗F_{ir}\\ M_{s}\left(F_{ir}^{\prime }\right) & =\sigma ({\mathrm{Conv}}^{7\times 7}\left(\left[\mathrm{AvgPool}\left(F_{ir}^{\prime }\right);\mathrm{MaxPool}\left(F_{ir}^{\prime }\right)\right]\right))\\ F_{ir}^{att} & =M_{s}\left(F_{ir}^{\prime }\right)⊗F_{ir}^{\prime } \end{array}$$

Second, in the visible light branch, to simulate human visual perception of textures at different granularities, the input feature $F_{vis}$ is pre-processed via 3×3 convolution and then input into the Multi-Scale Feature Aggregation block. This block employs four sets of convolution kernels with varying dimensions (1×1,3×3,5×5,7×7) in parallel to capture features ranging from fine textures to global contours. After concatenating the features from each scale, dimensionality reduction and integration are performed via a 1×1 convolution and ReLU activation to obtain the multi-scale feature $F_{vis}^{ms}$:

(7) $$F_{vis}^{ms}=\mathrm{ReLU}\left({\mathrm{Conv}}^{1\times 1}\left({\mathrm{Concat}}_{k\in \{1,3,5,7\}}\left({\mathrm{Conv}}^{k\times k}\left(F_{vis}\right)\right)\right)\right)$$

Finally, to compensate for potential information loss between visual and semantic features, the residual feature $F_{res}$ output by the KVA module is introduced into the fusion layer. The enhanced infrared feature $F_{ir}^{att}$, the multi-scale visible light feature $F_{vis}^{ms}$, and the semantic residual feature $F_{res}$ are concatenated in the channel dimension (Concat) and mapped non-linearly through a Multi-Layer Perceptron (MLP) to output the final fused feature $F_{s}$:

(8) $$F_{s}=\mathrm{MLP}\left(\mathrm{Concat}\left(F_{ir}^{att},F_{vis}^{ms},F_{res}\right)\right)$$

This module constructs a deep synergistic mechanism of physical perception and semantic enhancement. First, by integrating the channel–spatial attention mechanism within the infrared branch, the model effectively suppresses background noise in thermal imaging, thereby precisely focusing on critical regions reflecting internal qualitative changes in seeds. Simultaneously, the multi-scale convolution structure endows the model with robustness in adapting to texture differences across seeds of varying sizes by expanding the receptive field. More critically, the innovative introduction of the semantic residual feature $F_{res}$ overcomes the limitations of purely visual modalities. This ternary fusion strategy not only aggregates the dual advantages of infrared internal perspective and visible external texture but also effectively utilizes complementary semantic information that was not fully aligned. Consequently, this significantly enhances the completeness of feature representation and substantially improves the comprehensive performance of the model in joint variety and viability discrimination within complex agricultural scenarios.

#### 2.4.5. Joint-Learning Head Module

To achieve high-precision seed variety identification and internal viability detection simultaneously within a unified framework, the Joint-Learning Head Module (JLH) is designed. The motivation for this design stems from the necessity of decoupling task characteristics: seed variety identification relies predominantly on the consistent matching between apparent features and encyclopedic semantics, whereas viability detection depends primarily on physical feature discrepancies following the fusion of infrared and visible light. Consequently, the JLH module adopts a dual-branch parallel structure. Specifically, the semantically enhanced feature $F_{sem}$ output by the KVA module is utilized for metric-based variety inference, while the fused feature $F_{s}$ output by the DSF module is employed for distribution-based viability classification. Furthermore, the optimization process of both branches is coordinated through a multi-task loss function, thereby achieving comprehensive perception of seed traits.

Specifically, the module comprises a semantic matching branch and a viability classification branch. First, within the semantic matching branch, to fully leverage the zero-shot or few-shot generalization capabilities conferred by image-text pre-training, traditional fully connected classification layers are discarded in favor of a cosine similarity metric. The feature $F_{sem}$ output by the KVA module undergoes normalization (Norm) and is subsequently compared against the text embedding vector matrix $W_{text}$ of all predefined categories to calculate similarity. For the *i*-th category, the matching score $S_{i}$ is calculated as the dot product of the visual feature and the text feature:

(9) $$S_{i}=\frac{F_{sem}\cdot {\left(W_{text}^{\left(i\right)}\right)}^{⊤}}{||F_{sem}||\;||W_{text}^{\left(i\right)}||}$$

The generated similarity matrix (Match Score) directly reflects the probability that the current seed belongs to each category and is supervised using the cross-entropy loss $L_{cls}$. Second, in the viability classification branch, addressing the high-dimensional fused feature $F_{s}$ output by the DSF module, Global Average Pooling (GAP) is first employed to compress spatial dimensions and extract global feature vectors possessing rotational invariance. Subsequently, the features are mapped into a binary classification space (Healthy/Necrotic) through two layers of Multi-Layer Perceptrons (MLP) and a projection layer (Project), outputting a probability distribution:

(10) $$P_{via}=\mathrm{Softmax}\left(\mathrm{Project}\left(\mathrm{MLP}\left(\mathrm{GAP}\left(F_{s}\right)\right)\right)\right)$$

Here, the binary cross-entropy loss $L_{via}$ is adopted to guide viability discrimination. Finally, to balance the learning progress of the two tasks, the total objective function is defined as the weighted sum of both:

(11) $$L_{total}=\lambda L_{cls}+\left(1-\lambda \right)L_{via}$$

where λ serves as the balancing hyperparameter. The advantage of this module lies in its construction of a balancing mechanism between task decoupling and joint optimization. By introducing metric learning into the variety identification task, the model is liberated from rigid dependence on fixed category labels, enabling the flexible utilization of semantic information to handle long-tail or novel category data. Conversely, in the viability detection task, the classical deep feature mapping structure is retained to ensure the exhaustive mining of infrared perspective features. This dual-pronged design, in conjunction with the constraints of the multi-task joint loss, not only prevents overfitting on single tasks but also promotes the mutual enhancement of semantic and physical representations within the latent space by sharing the underlying feature extractors, thereby significantly improving the comprehensive discriminative performance of the overall model.

## 3. Results and Discussion

### 3.1. Experimental Setup

To ensure experimental fairness and reproducibility, all evaluations were performed on a unified hardware and software platform. The hardware infrastructure consisted of a workstation equipped with high-performance computing resources, including an Intel^®^ Core^™^ i9-12900K CPU @ 3.20 GHz processor, an NVIDIA GeForce RTX 3090 GPU (24 GB VRAM), and 64 GB of RAM. The software environment operated under the Ubuntu 20.04 LTS operating system. PyTorch 1.13.1 was selected as the deep learning framework, utilized in conjunction with CUDA 11.7 and CUDNN 8.5 for accelerated computing. Image processing and data augmentation primarily relied on the OpenCV and Albumentations libraries, while textual data preprocessing was accomplished using the HuggingFace Tokenizers library.

Regarding implementation specifics, both visible light and infrared images input into the network were uniformly resized to a resolution of 224×224 pixels and normalized. The training process employed the AdamW optimizer with momentum parameters set to $\beta_{1}=0.9$ and $\beta_{2}=0.999$, and a weight decay coefficient of $1\times 10^{-4}$. The batch size was established at 32, with a total of 100 training epochs. The initial learning rate was set to $1\times 10^{-4}$, utilizing a Cosine Annealing Strategy to dynamically adjust the learning rate down to a minimum lower bound of $1\times 10^{-6}$. Furthermore, to prevent overfitting, data augmentation strategies—including random cropping, horizontal flipping, and color jittering—were introduced during the training phase. For the balancing parameter λ within the multi-task loss function, a value of 0.6 was assigned to achieve optimal joint discriminative performance, as verified via grid search experiments.

To comprehensively evaluate the effectiveness of S3-Net, ResNet-50 [25], EfficientNet-B4 [26], DenseNet-121 [27], Vision Transformer (ViT-B/16) [28], Swin Transformer (Swin-T) [29], MoCo v2 [30], and CLIP (ViT-B/32) [31] were selected as comparative methods. All benchmark models were retrained or fine-tuned employing identical data splits and hyperparameter settings.

### 3.2. Evaluation Metrics

To comprehensively and objectively assess the performance of S3-Net in both seed variety identification and viability detection tasks, a multi-dimensional evaluation metric system was adopted in this study. Regarding fundamental classification performance, Accuracy, Precision, Recall, and the F1-score were selected as core metrics. Specifically, Accuracy measures the proportion of correct predictions across all test samples. Precision reflects the proportion of actual positive samples among those predicted as positive, which is critical for minimizing the false detection rate. Recall quantifies the proportion of all actual positive samples that were correctly identified, a metric of significant importance in agricultural screening for preventing the omission of inferior seeds. Finally, the F1-score, defined as the harmonic mean of Precision and Recall, serves to comprehensively evaluate model robustness under conditions of class imbalance. The calculation formulas for the aforementioned metrics are defined as follows:

(12) $$\begin{array}{cc} \mathrm{Accuracy} & =\frac{TP+TN}{TP+TN+FP+FN}\\ \mathrm{Precision} & =\frac{TP}{TP+FP},\;\mathrm{Recall}=\frac{TP}{TP+FN}\\ F1\text{-}\mathrm{score} & =2\times \frac{\mathrm{Precision}\times \mathrm{Recall}}{\mathrm{Precision}+\mathrm{Recall}} \end{array}$$

Here, TP, TN, FP, and FN represent the counts of true positive, true negative, false positive, and false negative samples, respectively. In addition to the fundamental metrics, the Area Under the Receiver Operating Characteristic Curve (AUC-ROC) was utilized as a supplementary metric for the binary seed viability classification task to assess the discriminative capability of the model across varying thresholds. An AUC value approaching 1.0 indicates a stronger capability to distinguish between healthy and necrotic seeds; furthermore, this metric exhibits robust resilience to variations in sample class distribution.

### 3.3. Comparison with State-of-the-Art Methods

To validate the comprehensive performance of S3-Net within complex agricultural scenarios, a systematic comparison was conducted between the proposed method and the previously mentioned comparative methods. All experiments were rigorously executed adhering to identical training strategies and five-fold cross-validation settings.

Table 3 presents the comparative results of the fine-grained seed species identification task against various strong baselines. Experimental data indicate that S3-Net outperforms the comparative benchmark models across all evaluation metrics, including CNNs, Transformers, and specialized multimodal frameworks. Among traditional Convolutional Neural Networks, EfficientNet-B4 achieved the best performance with an accuracy of 92.8 percent. Standard spectral classification approaches such as 1D-CNN showed the lowest accuracy (82.4 percent) because they rely primarily on chemical responses and lack the spatial resolution to capture morphological textures essential for variety identification. Furthermore, compared to general multimodal fusion frameworks like IFCNN (93.8 percent accuracy), S3-Net demonstrates a clear advantage by incorporating semantic guidance. This performance gap is primarily attributed to the local receptive field characteristics of standard convolution and the lack of high-level inductive bias in traditional fusion methods, which render them susceptible to confusion among closely related varieties. In contrast, S3-Net increased accuracy to 96.9 percent by introducing encyclopedic semantic priors. Compared to the Swin Transformer based on global attention mechanisms (94.2 percent accuracy) and the large-scale pre-training model CLIP (95.4 percent accuracy), our framework maintains superior discriminative power. Although S3-Net has a larger total parameter count of 165.4 M, the use of parameter-frozen backbone encoders ensures training efficiency while providing high-dimensional semantic representation. The KVA module explicitly maps domain-specific descriptions into the visual space, effectively correcting classification errors along ambiguous boundaries where appearances are extremely similar.

**Figure 6:**
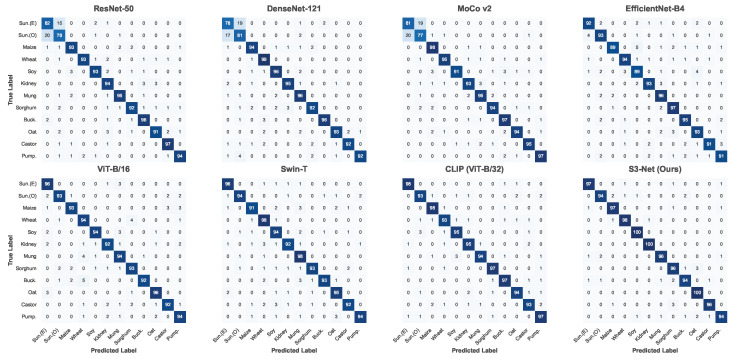
Visual comparison of confusion matrices across different models for the fine-grained seed species classification task. The horizontal axis represents the predicted classes, while the vertical axis denotes the ground truth classes.

Figure 6 visually presents the classification performance of the comparative methods in the fine-grained identification task involving twelve seed categories. An examination of the confusion matrices for the baseline models reveals distinct limitations within Convolutional Neural Networks, exemplified by ResNet-50 and DenseNet-121, when processing closely related varieties exhibiting highly similar morphological features. Specifically, significant confusion noise is observed in the off-diagonal regions between the two subspecies categories: Sunflower (Edible) and Sunflower (Oil). This indicates the difficulty encountered by pure vision models in establishing sufficiently discriminative boundaries based solely on texture differences. In contrast, the confusion matrix of S3-Net exhibits a pronounced diagonal clustering characteristic, with off-diagonal elements approaching zero. This performance enhancement is primarily attributed to the incorporation of the Knowledge–Vision Alignment module. By mapping morphological semantic descriptions from encyclopedic texts into the visual feature space, this module effectively expands the inter-class feature distance. Consequently, misclassifications along ambiguous visual boundaries are significantly rectified, verifying the error-correction capability of semantic priors in fine-grained classification tasks.

Table 4 presents the experimental results obtained by various models in the seed viability detection task. As evidenced by the performance of the baseline models, methods relying solely on visual input have already achieved a high level of proficiency. Specifically, the Swin-T and CLIP models achieved accuracies of 94.1% and 95.4%, respectively, with AUC metrics exceeding 96%. This indicates that, in the majority of instances, a strong statistical correlation exists between the viability state of seeds and their apparent characteristics (e.g., seed coat luster, plumpness, or minute shrinkage). Advanced feature extractors are capable of effectively capturing these visual cues for discrimination. Building upon this high-performance baseline, S3-Net achieved stable performance improvements, reaching an accuracy of 96.8% and increasing the AUC to 98.6%. Compared to CLIP, the best-performing single-modal model, S3-Net demonstrated an accuracy improvement of 1.4 percentage points. Although the numerical increment appears modest, achieving further enhancement within the high-accuracy range (>95%) typically encounters the challenge of diminishing marginal returns. The advantage of S3-Net is primarily manifested in its capability to rectify predictions for specific hard-to-distinguish samples. For samples exhibiting an intact appearance but suffering from slight internal mildew or necrosis, extracting effective features solely from visible light images proves difficult; however, infrared spectroscopy possesses physical sensitivity to changes in internal organic composition. By integrating this penetrating spectral information, the DSF module provides a necessary supplement to visual features. Consequently, while maintaining overall high precision, the reliability of the model’s judgment is further enhanced.

**Figure 7:**
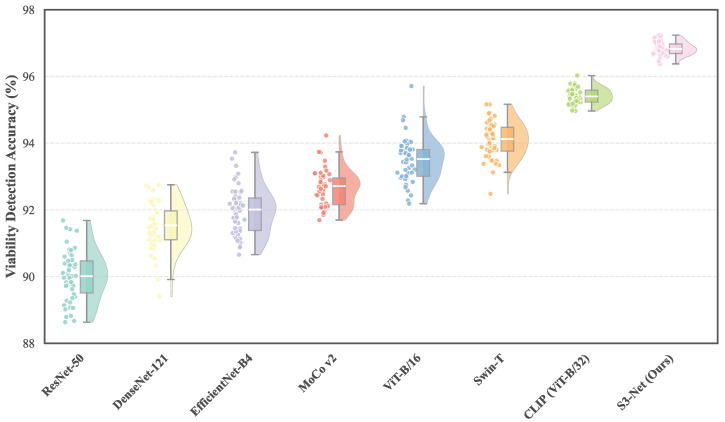
Performance distribution comparison of seed viability detection across different models.

As illustrated in Figure 7, Traditional CNN-based architectures, such as ResNet-50 and DenseNet-121, exhibit long-tail violin shapes with widely dispersed scatter points, indicating high variance and sensitivity to random initialization or data sampling. In contrast, S3-Net achieves a significant performance leap, with its distribution cluster positioned distinctly higher than all competing baselines, reaching a peak accuracy of approximately 96.8%. Crucially, S3-Net demonstrates exceptional stability, characterized by a highly compact box plot and minimal interquartile range. Even when compared to strong Transformer-based baselines like CLIP (ViT-B/32), our method maintains a tighter error margin, confirming that the synergistic fusion of spectral internal features and encyclopedic semantic priors effectively mitigates model uncertainty and ensures consistent, high-precision detection in practical agricultural scenarios.

**Figure 8:**
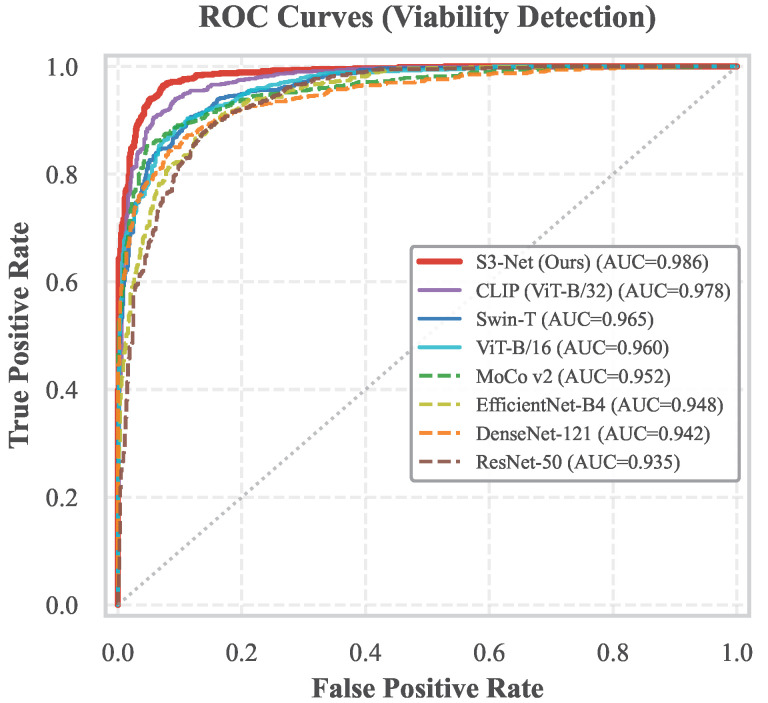
Comparison of Receiver Operating Characteristic (ROC) curves among different models for the seed viability detection task. The horizontal and vertical axes denote the False Positive Rate (FPR) and True Positive Rate (TPR), respectively.

Figure 8 illustrates the performance disparities between S3-Net and other benchmark models in the binary classification task of seed viability. Regarding the overall trend, the ROC curve of S3-Net (indicated by the solid red line) encompasses the curves of comparative methods across nearly all threshold settings. An AUC value of 0.986 is achieved, which significantly outperforms the second-best CLIP model (AUC = 0.978). Although a degree of intertwining among curves is observed in certain intermediate regions—reflecting variations in the sensitivity of different feature extractors to specific sample distributions—an examination of the magnified subplot in the upper-left corner reveals that S3-Net maintains the highest true positive detection rate within the low false positive rate interval. This interval is of critical importance in agricultural applications, where the minimization of misclassifying healthy seeds is prioritized. This performance advantage under stringent thresholds is primarily attributed to the incorporation of infrared physical features via the dual-spectral fusion module. This integration enables the model to sustain robust discriminative capability when identifying challenging samples with inconspicuous visible light features (e.g., slight internal mildew), thereby effectively surpassing the performance ceiling inherent to single visual modalities.

**Figure 9:**
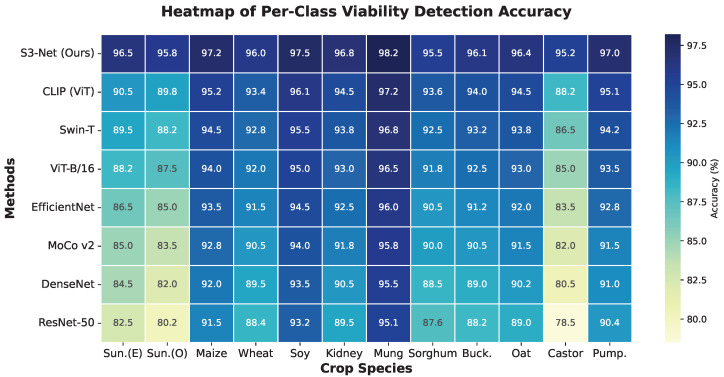
Heatmap of classification accuracy for different models across the viability detection task for 12 crop seed species. Each row represents a comparative method, while each column corresponds to a specific crop category. Numerical values and color intensity within the cells indicate detection accuracy (%), with darker hues denoting superior performance of the model on the respective category. This figure intuitively reflects differences in generalization capability and robustness among models across crops with varying seed coat characteristics.

Figure 9 visually presents the performance distribution of S3-Net alongside seven benchmark models across fine-grained categories via a heatmap. Regarding the overall color tone, the row corresponding to S3-Net maintains the highest color saturation across all crop columns, indicating strong cross-category robustness. A vertical comparison of detection difficulty across different crops reveals that single-modal vision models (e.g., ResNet-50, EfficientNet, and even ViT) exhibit significant performance degradation when processing categories such as Castor Bean and Sunflower (corresponding regions show markedly lighter colors, with accuracy dropping to the 80∼88% range). This phenomenon is attributed to the thick, rigid, and complexly textured seed coats characteristic of these species, which severely obscure the physiological state of the internal endosperm. Consequently, models relying solely on RGB apparent features struggle to extract effective discriminative evidence. In contrast, S3-Net maintains a high accuracy exceeding 95% on these long-tail hard samples. This performance advantage stems from the effective integration of the penetrative properties of short-wave infrared light by the Dual-Spectral Fusion module. This integration enables the model to directly perceive internal moisture content and organic lesions through the seed coat, thereby achieving precise inference even when visual features are restricted. Furthermore, for simple samples with thinner seed coats, such as Mung Bean, the performance disparities among models are relatively minor. This further confirms that the core contribution of S3-Net lies in addressing the challenge of non-destructive detection under conditions of complex occlusion.

### 3.4. Evaluation on Few-Shot Learning Scenarios

To further evaluate the generalization capability of S3-Net within data-scarce scenarios and to verify the efficacy of encyclopedic semantic priors embedded in the KVA module, a comparative experiment based on few-shot learning was designed. In practical agricultural applications, the acquisition and annotation of large-scale imagery for novel seed varieties are typically time-consuming and labor-intensive; consequently, the recognition performance of models under conditions of extremely limited samples possesses significant practical value. The experimental setup adhered to the standard *N*-shot protocol, wherein *N* samples (N∈{1,5,10,20}) were randomly selected from each category to constitute the training set, while the remaining samples served as the testing set. To mitigate the impact of random sampling, each experimental group was repeated five times, and the mean accuracy along with the standard deviation (Mean ± Standard Deviation) are reported.

Table 5 presents the recognition accuracy of various models under different sample size settings, with the experimental data exhibiting a distinct stratification of performance. Under extremely low-sample conditions (1-shot and 5-shot), all purely supervised vision models (including the CNN family members ResNet, EfficientNet, and DenseNet, as well as the Transformer family members ViT and Swin) demonstrated severe maladaptation. Even for Swin-T, which exhibited relatively superior performance, the 1-shot accuracy was merely 38.4% with a large standard deviation, indicating substantial model instability in the absence of sufficient data support. Although the self-supervised model MoCo v2 elevated the 1-shot accuracy to 45.6% by leveraging pre-trained feature invariance, a gap remains regarding practical utility standards. In contrast, the CLIP model, which incorporates semantic information, and the S3-Net proposed in this paper demonstrated significant robustness. S3-Net achieved an accuracy of 72.8% with only a single training sample, representing improvements of 36.6 and 35.3 percentage points compared to EfficientNet-B4 and ViT-B/16, respectively. This substantial performance gap reveals the propensity for overfitting in pure vision models lacking inductive bias: when samples are extremely scarce, these models tend to focus excessively on background noise or non-critical textures within the images. Furthermore, even when compared with CLIP, which similarly utilizes textual information, S3-Net maintained advantages of 4.6 and 3.9 percentage points in 1-shot and 5-shot settings, respectively. This advantage is primarily attributed to the cross-modal semantic constraint mechanism established by the KVA module. By utilizing high-level descriptions of seed morphology from encyclopedic texts (e.g., long oval, black stripes) as potent prior knowledge, visual features are guided to map accurately to corresponding semantic cluster regions within the feature space. Under conditions of visual sample scarcity, this strong supervision signal from the text domain effectively regularizes the feature manifold and suppresses feature distribution drift. Consequently, the deficiencies of purely data-driven learning are compensated for through the introduction of auxiliary supervision from domain knowledge, significantly enhancing the data utilization efficiency and generalization performance of the model.

**Figure 10:**
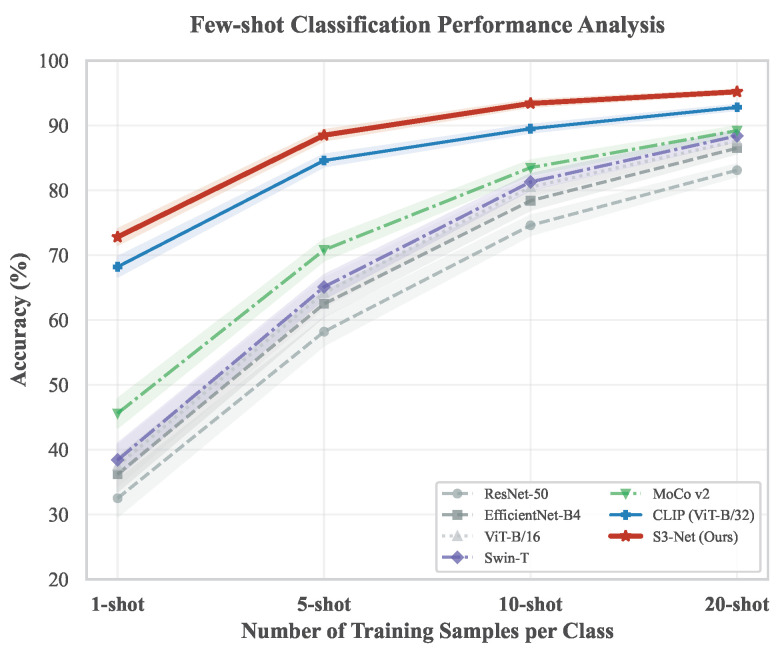
Comparison of classification performance trends between S3-Net and other benchmark models under few-shot settings. The horizontal axis represents the number of training samples per category (*N*-shot), while the vertical axis denotes the Accuracy. Shaded regions indicate the standard deviation range across five independent experiments. This figure highlights the significant performance advantage of S3-Net over pure vision models in extremely low-data regimes (e.g., 1-shot).

Figure 10 clearly illustrates the performance evolution of various models as the training sample size increases from extreme scarcity (1-shot) to relative sufficiency (20-shot). Experimental results exhibit distinct performance stratification characteristics. First, multi-modal models, represented by S3-Net and CLIP, demonstrate superior performance. Attributed to the incorporation of multi-modal semantic priors, the initial accuracy of both models exceeds 68% under the 1-shot setting. In particular, S3-Net, leveraging the injection of domain knowledge specific to agriculture, maintains a consistent advantage of approximately 4% over CLIP during the 1-shot and 5-shot stages. Furthermore, the significantly smaller standard deviation demonstrates exceptional robustness and stability. Second, the performance curve of the self-supervised model MoCo v2 lies between those of the multi-modal models and the purely supervised models. This result indicates that although unsupervised feature pre-training mitigates the dependence on labeled data to a certain extent, the resulting performance gains remain inferior to those yielded by explicit semantic enhancement strategies. Finally, pure vision fully supervised models, such as ResNet and Swin Transformer, exhibit severe maladaptation under conditions of extremely low sample availability, with 1-shot accuracies falling below 40%. Although the rate of performance improvement for these models is significant as the sample size increases, their performance at the 20-shot mark still fails to match the level achieved by S3-Net at only 5-shot. This significant performance gap strongly validates the effectiveness of the KVA module: by introducing encyclopedic knowledge as a strong Inductive Bias, the module successfully compensates for the information entropy deficit caused by the scarcity of visual samples, thereby conferring robust, human-like generalization capabilities upon the model under low-data conditions.

### 3.5. Impact of Multi-Spectral Fusion

To investigate the specific contributions of distinct spectral modalities to seed recognition tasks and to verify the effectiveness of the dual-spectral fusion (DSF) module in integrating heterogeneous features, a comprehensive ablation study was conducted. This experiment evaluated model performance by isolating variables, specifically assessing the exclusive use of the visible light (RGB) branch, the exclusive use of the infrared (IR) branch, and the deployment of different fusion strategies including early fusion (input-level concatenation), late fusion (decision-level averaging), and the proposed S3-Net ternary feature fusion. To ensure comparative fairness, all experiments were conducted under identical training parameters and KVA semantic enhancement settings.

Table 6 presents the performance disparities among different fusion strategies in the seed species identification task. Experimental data indicate that while the visible light modality plays a dominant role (91.5% accuracy) due to rich texture information, simple fusion methods offer limited gains. Early fusion achieves 93.0% accuracy but is hindered by the resolution discrepancy and pixel-level noise between sensors. Late fusion improves this to 94.1% by combining independent modal decisions, yet fails to capture the intricate spatial-spectral correlations. S3-Net achieves the highest accuracy of 96.9% by utilizing a ternary feature fusion strategy. This suggests that the deep integration of infrared features, multi-scale visible textures, and semantic residuals effectively corrects confusion errors between varieties with highly similar appearances.

Table 7 reveals a significant performance reversal where the IR modality outperforms RGB in viability detection. While early fusion (91.2% accuracy) and late fusion (92.5% accuracy) provide improvements over single modalities, they fall short of the S3-Net framework (95.8% accuracy). The significant gain in S3-Net is attributed to the attention mechanism of the DSF module, which utilizes visible light boundaries to suppress infrared thermal diffusion noise. This synergistic perception mode fundamentally resolves the reliability issues associated with standard fusion techniques in non-destructive internal quality assessment.

### 3.6. Ablation Study

To validate the effectiveness of the key components within the S3-Net architecture and to quantify the specific contributions of the Knowledge–Vision Alignment (KVA) module and the Dual-Spectral Fusion (DSF) module to the final performance, a series of ablation experiments were conducted based on the test set. The experiments utilized ResNet-50, containing only the visible light branch, as the Baseline model. Variant models were constructed by selectively removing or retaining specific components. The specific settings are defined as follows: w/o DSF indicates the retention of the KVA module but the removal of the Dual-Spectral Fusion module; w/o KVA indicates the retention of the DSF module but the removal of the semantic alignment module; and S3-Net (Full) represents the complete model containing all components. This design aims to decouple the underlying mechanisms of semantic enhancement and physical perception across different tasks.

Table 8 presents the performance of each model variant on the seed species identification task. Experimental data indicate that the KVA module plays a decisive role in enhancing fine-grained classification performance. A comparison between the Baseline and the w/o DSF variant (i.e., with KVA semantics introduced) reveals that the model’s accuracy leaped from 91.5% to 96.2%, achieving a significant increase of 4.7 percentage points, while the F1-score also improved by 4.8 percentage points. This result confirms the effectiveness of semantic priors: basic vision models often suffer from confusion in the feature space when processing closely related varieties with highly similar appearances. In contrast, the KVA module injects high-level semantic descriptions (such as textual definitions of shapes and textures) into visual features through cross-modal alignment, widening the distance between different categories on the feature manifold, thereby effectively correcting misclassifications along ambiguous boundaries. Conversely, the accuracy of the w/o KVA variant (i.e., with only infrared features introduced) improved only to 92.8%, indicating that the gain provided by infrared spectroscopy in distinguishing external morphological features is far less than the guidance provided by semantic information. Ultimately, S3-Net combined the advantages of both, reaching the highest accuracy of 96.9%.

Table 9 further reveals the contributions of each component in the seed viability detection task, presenting a trend distinct from that of species identification. In this task, the DSF module demonstrated irreplaceable core value. The accuracy of the Baseline was only 71.2%, indicating that internal viability is difficult to accurately determine based solely on RGB appearance. The performance improvement of the w/o DSF variant, which introduced the KVA module, was negligible (+1.3%), suggesting that semantic knowledge cannot compensate for the absence of physical information. However, once the DSF module was introduced (refer to the w/o KVA variant), accuracy surged to 94.5%, and the AUC metric improved by 22.5 percentage points. This leap in performance is explained at the physical level: loss of viability is typically accompanied by the degradation of internal organic components or moisture loss; these changes are latent in visible light but exhibit significant spectral responses in the infrared band. The DSF module effectively extracted these critical physical features through attention mechanisms. Furthermore, the accuracy of the complete S3-Net model further improved to 95.8%, indicating that precise species identification (contributed by KVA) provides category-specific prior context for viability discrimination. The synergistic operation of both ultimately achieved precise, non-destructive assessment of seed vitality.

**Figure 11:**
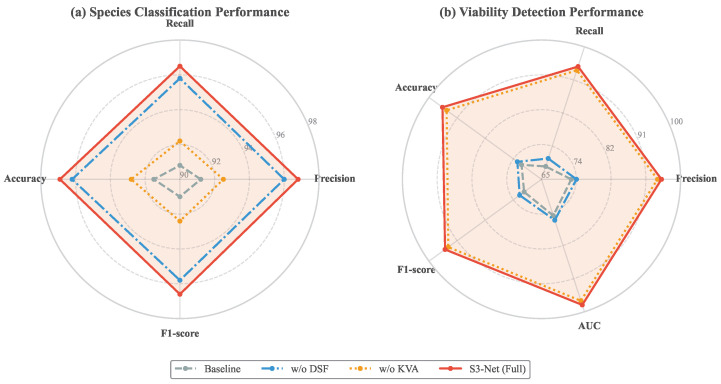
Visual comparison of multi-dimensional performance in ablation studies using radar charts: (**a**) illustrates model performance in the fine-grained seed species identification task; (**b**) illustrates model performance in the seed viability detection task. The five radial axes represent different evaluation metrics (Precision, Recall, Accuracy, F1-score, and AUC), while the enclosed polygonal regions of different colors reflect the comprehensive performance coverage of the baseline model (Baseline), its variants (w/o DSF, w/o KVA), and the complete model (S3-Net).

Figure 11 intuitively presents the contributions of the KVA and DSF modules across different tasks through the topological morphological differences observed in the radar charts. In the species identification task depicted in Figure 11a, the variant model incorporating the KVA module (w/o DSF, blue outline) exhibits a significant expansion in area compared to the baseline model (Baseline, gray outline), with its performance boundaries approaching those of the complete S3-Net. This phenomenon indicates that when addressing fine-grained classification problems characterized by highly similar appearances, semantic priors derived from encyclopedic texts play a dominant role in feature enhancement, whereas physical information provided by infrared spectroscopy serves merely as an auxiliary supplement. Conversely, a distinctly different pattern of performance distribution is observed in the viability detection task shown in Figure 11b. Models relying solely on vision and semantics (Baseline and w/o DSF) are constrained by the physical limitation that visible light cannot penetrate the seed coat; consequently, their performance metrics are confined to the low-value region around 70% (inner circle). However, upon the introduction of the DSF module (w/o KVA, orange outline), model performance undergoes a leap, rapidly expanding to the high-value region above 95% (outer circle). This strongly confirms the decisive value of short-wave infrared spectroscopy in detecting internal latent physiological lesions. S3-Net (red filled area) achieves the maximum envelope area in both figures, demonstrating that optimal comprehensive performance balance is attained in multi-task synergy through the integration of semantic guidance and physical perception.

### 3.7. Robustness Analysis Against Artificial Aging Artifacts

To address concern regarding potential feature leakage caused by macroscopic surface changes such as wrinkling or darkening during artificial accelerated aging, we conducted a targeted evaluation to determine whether the high performance of S3-Net relies on artificial artifacts or genuine internal physiological signals. We partitioned the non-viable test samples into two distinct subsets: the Artificial Aging (AA) group, which was subjected to high-temperature and high-humidity treatment (45 °C, 95% RH, 72 h) and often exhibited visible surface modifications, and the Natural Aging (NA) group, derived from long-term warehouse storage, which appeared morphologically intact and visually indistinguishable from healthy seeds. We evaluated the viability detection accuracy and AUC of the RGB-only branch, the IR-only branch, and the S3-Net across these two groups to quantify the reliance on surface versus internal features.

The experimental results presented in Table 10 reveal a significant performance discrepancy for the RGB-only model. While the RGB branch achieves high accuracy (90.5%) on the AA group by exploiting visible surface artifacts, its performance collapses to 64.2% on the NA group where such artifacts are absent. This confirms the suspicion that purely visual models are susceptible to feature leakage when trained on artificially aged data. In contrast, the IR-only branch and the S3-Net framework demonstrate remarkable stability; the IR-only model maintains an accuracy of 88.5% on natural samples with a negligible drop of only 1.3 percentage points compared to the artificial group, while S3-Net achieves an even higher accuracy of 95.4% on the NA group. This indicates that while RGB features provide auxiliary “short-cuts” in artificial scenarios, SWIR-based internal sensing remains the core modality for capturing true physiological degradation, such as lipid oxidation and moisture redistribution, ensuring reliability in real-world applications where seeds may not exhibit obvious surface lesions.

## 4. Discussion

### 4.1. Semantic Enhancement Mechanism

**Figure 12:**
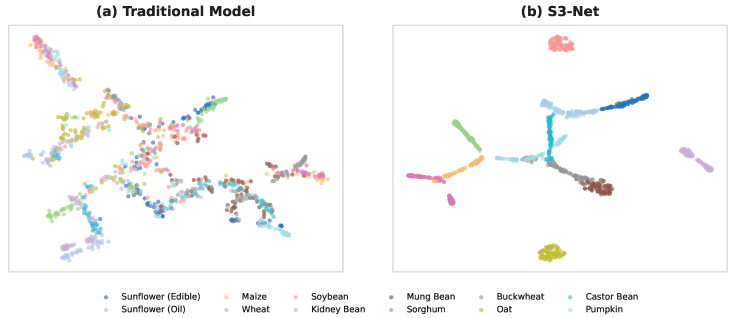
t-SNE visualization of feature distributions on the test set. (**a**) Features extracted by traditional model, showing significant overlap between closely related categories. (**b**) Features extracted by S3-Net, demonstrating clear decision boundaries and compact intra-class clustering. This comparison validates that the injection of semantic priors effectively reconstructs the feature manifold, enhancing discriminability for visually similar seeds.

In the seed species identification task, S3-Net demonstrated performance significantly superior to those of traditional vision models, particularly exhibiting strong robustness in few-shot scenarios. To intuitively analyze the internal mechanism of this performance enhancement, the t-SNE algorithm was utilized to visualize the dimensionality reduction in high-dimensional features from test samples, as shown in Figure 12. The visualization results clearly reveal how the KVA module bridges the semantic gap between low-level visual features and high-level semantic concepts. As illustrated in Figure 12a, traditional deep learning models rely primarily on data-driven approaches, tending to distinguish samples by memorizing texture biases within the training set [32]. However, due to the extremely high inter-class similarity of crop seeds, different sunflower subspecies are nearly indistinguishable in terms of pixel-level texture [33]. Consequently, pure vision models form substantial overlapping regions within the feature space, resulting in extremely blurred decision boundaries. In contrast, Figure 12b displays the feature distribution generated by S3-Net, characterized by clear inter-class boundaries and compact intra-class distributions. This qualitative transformation is attributed to the fact that the encyclopedic text introduced by S3-Net serves not merely as labels but as a potent Inductive Bias. By mapping explicit morphological descriptions, such as oval shape and black stripes, into the visual embedding space, the model is compelled to focus on essential features with biological significance rather than background noise. This domain-specific alignment enables the model to possess human-like cognitive reasoning capabilities—specifically, constructing a feature manifold with stronger discriminative power by understanding species definitions rather than merely matching image patterns.

### 4.2. Physical Basis of Spectral Synergy

Experimental results on viability detection confirm that the incorporation of Short-Wave Infrared (SWIR) information is critical for breaking through the ceiling of visual detection. From the perspective of spectral physics, the loss of seed viability is typically accompanied by changes in cell membrane permeability, oxidation of internal oils, or a reduction in enzyme activity. Visible light imaging (RGB) responds only to the spectral reflectance of the object surface (380–780 nm), remaining in a physical blind spot regarding endosperm lesions concealed by the seed coat [34]. In comparison, photons in the SWIR band (900–1700 nm) possess stronger tissue penetration capabilities, and specific wavelengths can be resonantly absorbed by water molecules (O-H bonds) and organic matter (C-H bonds). The DSF module of S3-Net exploits this physical property: The physiological condition of the seed embryo, such as mildew or hollowing, differs significantly from that of a normally viable seed in terms of infrared spectral absorption characteristics. By learning these infrared spectral features, the model can infer the physiological state of the embryo, and consequently, the overall seed vigor. However, reliance solely on infrared images results in a loss of spatial resolution. Ablation experiments indicate that the DSF module, through an attention mechanism, effectively integrates the high-frequency textures of visible light (providing clear physical boundaries) with the spectral features of infrared light (providing internal physiological status) in a complementary manner. This synergistic sensing mode fundamentally resolves the reliability issues associated with single-modal techniques in non-destructive detection.

### 4.3. Mechanistic Interpretation of the Multimodal Framework

The superior performance of S3-Net stems from its ability to mimic the multidimensional cognitive process of human experts by synergizing visual, spectral, and semantic information. The framework improves classification and detection through two primary mechanisms. First, the Knowledge–Vision Alignment (KVA) module functions as a semantic anchor. By mapping descriptive encyclopedic priors into the visual feature space, it imposes a high-level logical constraint on the embedding manifold. This is particularly effective for fine-grained species identification, where visual textures of closely related varieties (e.g., different subspecies of sunflower) are nearly identical. The semantic features provide the necessary “discriminative logic” to resolve visual ambiguities. Second, the Dual-Spectral Fusion (DSF) module addresses the physical limitations of single-modal sensing. Visible light (RGB) provides high-resolution structural and boundary information, whereas short-wave infrared (SWIR) penetrates the seed coat to capture the physiological state of the embryo.

The relative contributions of these features are task-dependent. In species identification, semantic features contribute the most to the performance gain, as evidenced by the significant drop in accuracy when the KVA module is removed. These features act as a regularization mechanism that prevents the model from overfitting to superficial pixel-level noise. Conversely, in seed vigor detection, the spectral features from the infrared branch play a dominant role. Infrared spectroscopy is sensitive to moisture distribution and organic matter degradation within the seed embryo, signals that are entirely absent in the RGB domain. The multimodal framework essentially allows for a “divide-and-conquer” approach: vision provides the “where” (spatial localization), while semantics and spectra provide the “what” (varietal purity) and “how” (physiological viability), respectively.

### 4.4. Practical Application Value

**Figure 13:**
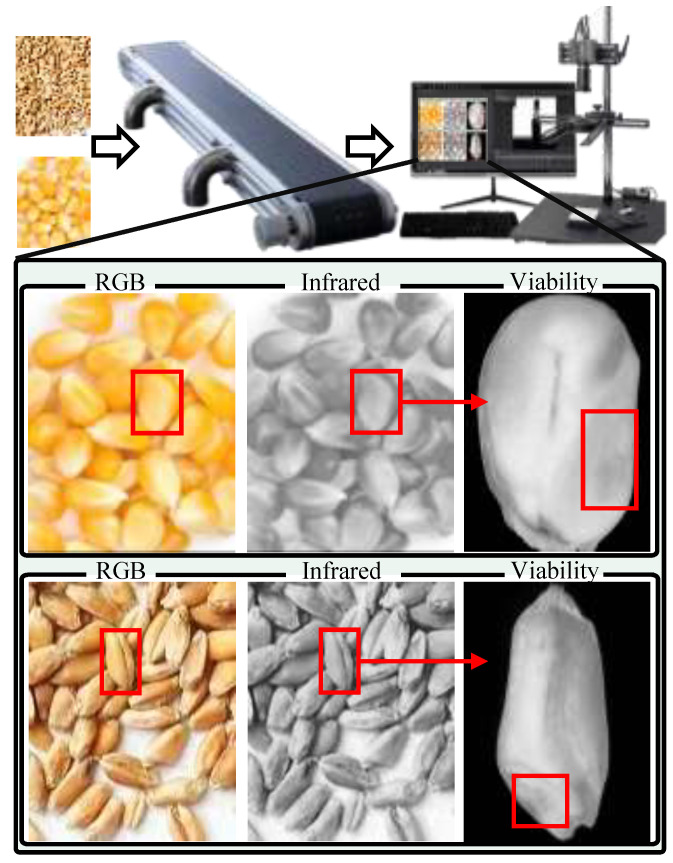
Visualization of seed vigor assessment by the S3-Net framework. The model analyzes and evaluates infrared spectral characteristics of the internal seed structure, with particular emphasis on the embryo region, enabling the assessment of embryonic physiological activity and subsequent inference of overall seed viability. The infrared spectral features of the seed embryo are intuitively visualized, and the key regions associated with embryo vitality are highlighted with red bounding boxes, facilitating manual inspection and verification of the model’s reliability.

As shonw in Figure 13, the proposal of S3-Net holds transformative implications for real-world agricultural phenotyping and industrial seed processing. Beyond high accuracy, the framework’s robust few-shot learning capability fundamentally changes the economic landscape of model deployment. In germplasm conservation and modern breeding, new varieties emerge at a rate that traditional large-scale data annotation cannot match. S3-Net reduces the “entry barrier” for AI adoption by allowing systems to learn from minimal samples combined with existing botanical documentation, effectively digitizing the expertise of veteran agronomists into an automated workflow.

**Figure 14:**
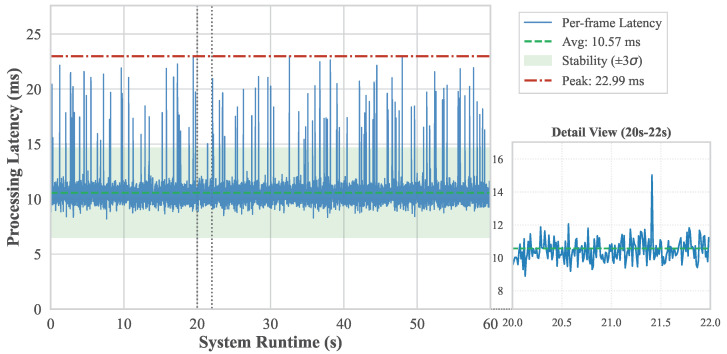
Runtime stability analysis of the S3-Net system over a continuous 60 s operation.

**Figure 15:**
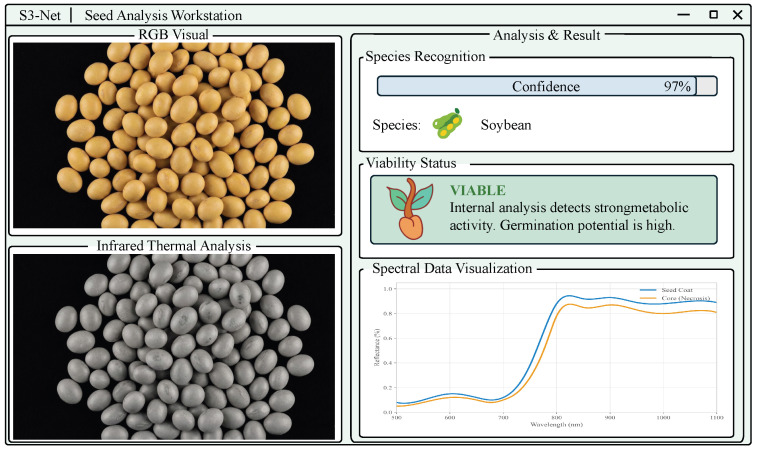
Visualization of the proposed S3-Net Intelligent Seed Analysis Workstation interface.

From a throughput perspective, the sustained performance of 95 fps—corresponding to approximately 500 seeds per second and achieved with a Batch Size of 1 to ensure real-time sequential processing while consuming only 4.8 GB of VRAM—aligns with the operational speeds of commercial gravity separators and optical sorters. This computational efficiency ensures that S3-Net can be integrated into high-speed sorting lines without creating a bottleneck. As illustrated in Figure 14, the narrow stability band and capped peak latency (22.99 ms) provide the deterministic timing required for real-time PLC (Programmable Logic Controller) synchronization in industrial ejector systems. Furthermore, the transition from destructive sampling to a non-invasive, 100%-inspection solution has profound economic consequences. By eliminating inferior or mislabeled seeds at the batch level prior to sowing, the framework minimizes the “waste” of land, water, and fertilizer on non-viable seeds, directly enhancing field emergence rates and ensuring food security. The workstation prototype shown in Figure 15 demonstrates that this complex multimodal sensing can be encapsulated into a user-friendly interface suitable for field deployment by non-technical personnel.

### 4.5. Physical Basis of SWIR Penetration in Dense Seed Coats

Regarding the concerns of optical attenuation in varieties with dense seed coats (e.g., castor and pumpkin seeds), it is essential to distinguish between direct transmittance and diffuse reflectance. In our system, the captured SWIR signal is primarily composed of diffuse reflectance photons that have undergone multiple scattering events within the internal tissues. While the hard, lignified seed coat indeed induces absorption and scattering, the lower refractive index contrast in the SWIR range compared to the visible range results in a higher “effective penetration depth” ($\delta_{eff}$), typically ranging from 1 to 3 mm for oily seeds.

Our results indicate that the S3-Net detects physiological signals directly from the embryo rather than secondary surface symptoms. This is evidenced by the fact that internal mildew or cavities in castor seeds often show distinct spectral absorption peaks at 1450 nm (water absorption) even when the seed coat remains structurally intact and visually normal. The DSF module leverages these deep-tissue spectral signatures, which provide a contrast ratio ($C_{internal}/C_{surface}$) significantly higher than that achievable with RGB imaging, thereby validating the physical assumption of internal sensing.

### 4.6. Strategic Advantages of Integrating Textual Semantics

The integration of botanical textual descriptions into the S3-Net framework offers three fundamental advantages for machine learning-based seed analysis. First, it achieves Semantic Anchoring and Domain Knowledge Injection. While purely visual models must infer discriminative features from raw pixels, the inclusion of encyclopedic priors allows the model to map visual regions to high-level biological concepts (e.g., “conical embryo,” “striated seed coat”). This guidance is crucial for resolving ambiguities in fine-grained classification where inter-class morphological variance is minimal. Second, language-vision alignment significantly enhances Few-shot Generalization. In scenarios with limited training samples, the textual descriptors act as a “knowledge bridge,” enabling the model to leverage universal physical attributes learned during the large-scale pre-training of the text encoder. This allows S3-Net to recognize novel varieties by aligning their visual features with known semantic descriptors. Third, the use of natural language improves model Interpretability. By calculating cross-modal attention, the system provides a “reasoning path” that shows which descriptive tokens (e.g., “darkening,” “wrinkling”) influenced the final viability or species prediction.

### 4.7. Agricultural–Economic Impact Analysis

From an agricultural–economics perspective, the proposed AI-driven multimodal sensing framework demonstrates substantial practical value across the seed industry supply chain. By enabling high-throughput and non-destructive viability detection, the system significantly reduces reliance on labor-intensive manual inspection, thereby lowering operational costs and improving screening consistency. Accurate discrimination between viable and non-viable seeds further mitigates the risk of yield loss caused by low-quality germplasm entering the planting stage. Moreover, fine-grained varietal identification supports optimized germplasm allocation and precision seeding strategies, which are critical for enhancing land-use efficiency and stabilizing crop productivity under large-scale cultivation. The integration of spectral sensing and semantic perception also enables early detection of latent physiological defects, reducing downstream storage and logistics losses. Importantly, the strong few-shot generalization capability of the framework lowers the data and infrastructure barriers for adopting intelligent sensing technologies in resource-constrained agricultural regions. From a macroeconomic standpoint, the deployment of automated seed quality sensing systems contributes to improved input–output efficiency, risk control, and digital transformation of the seed industry. By bridging intelligent perception technologies with agricultural production economics, the proposed framework provides scalable technological support for precision agriculture development and long-term sustainability of agri-food systems.

### 4.8. Limitations and Future Work

Despite the encouraging performance demonstrated in this study, several limitations should be acknowledged. First, although the proposed method was modeled to reflect industrial applications of seed inspection, it remains an abstraction of real-world conditions. While simplifying the experimental process, this abstraction cannot fully capture the complexity inherent in actual production environments. Specifically, the image dataset was collected under strictly controlled illumination to minimize the influence of ambient light and noise, whereas lighting conditions in operational seed processing facilities are inherently more variable and difficult to regulate. Second, although the simulated seed flow rate is representative of industrial throughput, the field of view of the imaging system is smaller than that typically used in large-scale industrial seed processing lines, which require broader imaging coverage. Third, while seed distribution on the conveyor belt was carefully controlled to theoretically avoid stacking, partial occlusions may still occur in real production scenarios due to irregular motion or accumulation, which could reduce model performance during actual deployment. Moreover, the current viability assessment is limited to a binary classification of “viable/non-viable” and does not yet address continuous vigor grading (e.g., high, medium, low vigor) required in agricultural production, thereby constraining its depth of application in precision breeding.

To address these limitations, future work will focus on deploying and validating the system under real industrial conditions. In terms of model refinement, images will be directly collected from operational seed processing facilities and systematically analyzed to validate and optimize the proposed model. A key objective will be to enhance the model’s robustness against environmental disturbances, illumination variations, and mild inter-seed occlusions. In terms of system modeling, the abstracted workflow will be concretized to match actual factory setups, ultimately enabling reliable deployment in open, unconstrained production environments. Additionally, existing datasets will be expanded by incorporating finer physiological and biochemical indicators, extending the current binary classification to continuous vigor grading prediction, thereby more comprehensively meeting the practical demands of precision agriculture for high-throughput, multi-dimensional seed quality assessment.

## 5. Conclusions

This study proposes an AI-driven multimodal sensing framework, termed the Semantic–Spectral Synergistic Sensing Network (S3-Net), to address two fundamental challenges in intelligent seed phenotyping: the fine-grained discrimination of visually similar varieties and the non-destructive assessment of internal physiological viability. By integrating encyclopedic semantic priors with dual-spectral physical perception, the framework establishes a unified sensing paradigm that overcomes the representational limitations of conventional single-modal vision approaches. Specifically, the Knowledge–Vision Alignment (KVA) module transforms domain-specific textual knowledge into semantic constraints that guide visual feature learning, substantially enhancing robustness under limited data conditions. Meanwhile, the Dual-Spectral Fusion (DSF) module exploits the penetrative sensing capability of short-wave infrared (SWIR) imaging in conjunction with high-resolution RGB texture perception, enabling the comprehensive characterization of both external morphology and embryo-related internal vitality. Extensive experiments conducted on the constructed multimodal sensing dataset demonstrate that S3-Net achieves classification accuracies of 96.9% for seed species identification and 95.8% for viability detection, consistently outperforming state-of-the-art models such as CLIP and Swin Transformer. Under extreme one-shot conditions, the proposed framework improves recognition accuracy by 40.3% compared with ResNet-50, highlighting its strong generalization capability. Furthermore, the system maintains a stable processing throughput of 95 fps, significantly exceeding the minimum operational requirement for industrial seed conveyance rates. Overall, the proposed S3-Net and its integrated sensing system provide a scalable and deployable solution for high-throughput, non-destructive seed quality inspection. More broadly, this work advances the development of intelligent sensor systems that fuse heterogeneous perception, semantic cognition, and autonomous analytics, offering a practical technological paradigm for future AI-driven sensing and precision agriculture applications.

## Figures and Tables

**Table 1:** Multimodal sensor configuration and data scale statistics for the proposed intelligent seed sensing system.

Sensor Type	Device Model/Specifications
RGB Vision Sensor	Hikvision MV-CA050-20GC; 2448 × 2048 resolution
Short-Wave Infrared Spectral Sensor	Xenics Bobcat-320; spectral range 900–1700 nm
Illumination Sensing System	Halogen tungsten full-spectrum light source for uniform imaging
Trigger Synchronization Module	Hardware-triggered synchronization with microsecond-level alignment
Conveyor Motion Sensor	Industrial encoder for flow rate and throughput monitoring

**Table 2:** Statistics of the collected multimodal seed dataset. The dataset includes 12 categories with a total of 6005 samples, showing a natural distribution between viable and non-viable classes.

Class ID	Category Name	Total Samples	Viable	Non-Viable
C01	Sunflower (Edible)	495	255	240
C02	Sunflower (Oil)	512	268	244
C03	Maize (Jinshan)	508	272	236
C04	Wheat (Yongliang)	486	238	248
C05	Soybean	503	261	242
C06	Red Kidney Bean	492	248	244
C07	Mung Bean	515	285	230
C08	Sorghum	488	250	238
C09	Buckwheat	497	246	251
C10	Oat	506	264	242
C11	Castor Bean	485	230	255
C12	Pumpkin Seed	518	260	258
Total	12 Classes	6005	3077	2928

**Table 3:** Performance comparison of seed species classification on the test set. Values are reported as Mean ± Standard Deviation (%). The best results are highlighted in bold. * and ** indicate p<0.05 and p<0.01 compared with the second-best model (CLIP), respectively.

Method	Params (M)	Precision	Recall	Accuracy	F1-Score
1D-CNN	1.2	81.8±0.9	82.0±0.8	82.4±0.8	81.5±0.9
ResNet-50	25.6	91.2±0.5	90.8±0.6	91.5±0.5	91.0±0.6
EfficientNet-B4	19.3	92.5±0.4	92.1±0.5	92.8±0.4	92.3±0.5
DenseNet-121	8.0	91.8±0.5	91.5±0.6	92.0±0.5	91.6±0.5
ViT-B/16	86.6	93.4±0.4	93.0±0.5	93.6±0.4	93.2±0.4
Swin-T	28.3	94.1±0.3	93.8±0.4	94.2±0.3	93.9±0.4
MoCo v2	25.6	92.0±0.6	91.8±0.7	92.3±0.6	91.9±0.6
IFCNN	32.4	93.5±0.5	93.2±0.6	93.8±0.5	93.3±0.5
CLIP (ViT-B/32)	151.2	95.2±0.3	94.9±0.3	95.4±0.3	95.0±0.3
**S3-Net (Ours)**	**165.4**	96.8±0.2*	96.5±0.3*	96.9±0.2**	96.6±0.2**

**Table 4:** Performance comparison of seed viability detection. Values are reported as Mean ± Standard Deviation (%). The best results are highlighted in bold. * and ** indicate p<0.05 and p<0.01 compared with the second-best model (CLIP), respectively.

Method	Precision	Recall	F1-Score	Accuracy	AUC
ResNet-50	90.5±0.8	89.2±1.0	89.8±0.9	90.2±0.8	93.5±0.7
EfficientNet-B4	92.1±0.6	91.5±0.8	91.8±0.7	92.0±0.7	94.8±0.5
DenseNet-121	91.4±0.7	90.8±0.9	91.1±0.8	91.5±0.8	94.2±0.6
ViT-B/16	93.5±0.5	92.8±0.6	93.1±0.5	93.4±0.6	96.0±0.4
Swin-T	94.2±0.4	93.6±0.5	93.9±0.5	94.1±0.5	96.5±0.4
MoCo v2	92.8±0.6	92.1±0.7	92.4±0.6	92.6±0.6	95.2±0.5
CLIP (ViT-B/32)	95.5±0.3	94.9±0.4	95.2±0.3	95.4±0.3	97.8±0.2
**S3-Net (Ours)**	96.8±0.2**	96.4±0.3*	96.6±0.2**	96.8±0.2**	98.6±0.1**

**Table 5:** Accuracy (%) comparison under few-shot learning settings. Values are reported as Mean ± Standard Deviation across 5 independent runs. The Full Data column serves as the upper-bound reference. Best results are highlighted in bold. * and ** indicate p<0.05 and p<0.01 compared with CLIP (ViT-B/32), respectively.

Method	Number of Shots (*N*)				Full Data
	1-Shot	5-Shot	10-Shot	20-Shot	
ResNet-50	32.5±2.8	58.2±2.1	74.6±1.5	83.1±0.9	91.5
EfficientNet-B4	36.2±2.6	62.5±1.9	78.4±1.4	86.5±0.8	92.8
DenseNet-121	34.8±2.7	60.1±2.0	76.2±1.5	84.8±0.9	92.0
ViT-B/16	37.5±3.1	64.2±2.2	80.5±1.3	87.6±0.8	93.6
Swin-T	38.4±2.5	65.1±1.8	81.3±1.2	88.4±0.8	94.2
MoCo v2	45.6±2.2	70.8±1.6	83.5±1.1	89.2±0.7	92.3
CLIP (ViT-B/32)	68.2±1.5	84.6±0.9	89.5±0.6	92.8±0.4	95.4
**S3-Net (Ours)**	72.8±1.2*	88.5±0.8**	93.4±0.5**	95.2±0.3**	96.9**

**Table 6:** Ablation study on seed species classification using different fusion strategies. Values are reported as Mean ± Standard Deviation (%). The results indicate that feature-level ternary fusion provides the most significant enhancement for variety discrimination. * and ** indicate p<0.05 and p<0.01 compared with Late Fusion, respectively.

Fusion Strategy/Modality	Precision	Recall	Accuracy	F1-Score
Visible Spectrum (RGB)	91.2±0.5	91.0±0.6	91.5±0.5	91.0±0.6
Infrared Spectrum (IR)	81.8±0.9	82.0±0.8	82.4±0.8	81.5±0.9
Early Fusion (Input-level)	92.6±0.4	92.3±0.5	93.0±0.4	92.5±0.5
Late Fusion (Decision-level)	93.8±0.4	93.5±0.5	94.1±0.4	93.7±0.5
S3-Net (Ternary Feature Fusion)	96.8±0.2*	96.5±0.3*	96.9±0.2**	96.6±0.2**

**Table 7:** Ablation study on seed viability detection using different fusion strategies. Values are reported as Mean ± Standard Deviation (%). The results highlight the superiority of the DSF module in handling spectral-spatial integration. * and ** indicate p<0.05 and p<0.01 compared with Late Fusion, respectively.

Fusion Strategy/Modality	Precision	Recall	Accuracy	F1-Score	AUC
Visible Spectrum (RGB)	72.5±1.2	68.4±1.5	71.2±1.2	70.4±1.3	74.6±1.0
Infrared Spectrum (IR)	89.2±0.7	88.5±0.8	89.5±0.6	88.8±0.7	91.5±0.5
Early Fusion (Input-level)	90.5±0.6	90.1±0.7	91.2±0.6	90.3±0.7	93.4±0.6
Late Fusion (Decision-level)	91.8±0.5	91.4±0.6	92.5±0.5	91.6±0.5	94.8±0.4
S3-Net (Ternary Feature Fusion)	95.2±0.4*	94.8±0.5*	95.8±0.4**	95.0±0.4**	98.2±0.2**

**Table 8:** Ablation results on seed species classification. “w/o” denotes the removal of a specific module from the full S3-Net. The Baseline represents the network without both KVA and DSF modules. * and ** indicate p<0.05 and p<0.01 compared with the Baseline, respectively.

Model Variant	Precision	Recall	Accuracy	F1-Score
Baseline (w/o KVA & DSF)	91.2±0.5	90.8±0.6	91.5±0.5	91.0±0.6
w/o DSF	96.0±0.3	95.8±0.4	96.2±0.3	95.8±0.3
w/o KVA	92.5±0.4	92.2±0.5	92.8±0.4	92.4±0.5
S3-Net (Full)	96.8±0.2**	96.5±0.3*	96.9±0.2**	96.6±0.2**

**Table 9:** Ablation results on seed viability detection. The comparison between “w/o KVA” and the Baseline highlights the critical role of the DSF module and infrared features for internal defect detection. * and ** indicate p<0.05 and p<0.01 compared with the Baseline, respectively.

Model Variant	Precision	Recall	Accuracy	F1-Score	AUC
Baseline (w/o KVA & DSF)	72.5±1.2	68.4±1.5	71.2±1.2	70.4±1.3	74.6±1.0
w/o DSF	73.8±1.0	70.5±1.2	72.5±1.0	71.8±1.1	75.8±0.9
w/o KVA	94.2±0.5	93.8±0.6	94.5±0.5	94.0±0.5	97.1±0.3
S3-Net (Full)	95.2±0.4*	94.8±0.5*	95.8±0.4**	95.0±0.4**	98.2±0.2**

**Table 10:** Performance comparison of viability detection on Artificial Aging (AA) and Natural Aging (NA) samples. Values are reported as Accuracy/AUC (%).

Evaluation Group	RGB-Only	IR-Only	S3-Net (Fusion)
Artificial Aging (AA)	90.5/92.4	89.8/91.5	96.2/98.4
Natural Aging (NA)	64.2/68.5	88.5/90.8	95.4/98.0
Performance Drop	−26.3/−23.9	−1.3/−0.7	−0.8/−0.4

## Data Availability

The data presented in this study are available on request from the corresponding author.
